# Hodological organization of spoken language production and singing in the human brain

**DOI:** 10.1038/s42003-023-05152-y

**Published:** 2023-07-26

**Authors:** Anni Pitkäniemi, Teppo Särkämö, Sini-Tuuli Siponkoski, Sonia L. E. Brownsett, David A. Copland, Viljami Sairanen, Aleksi J. Sihvonen

**Affiliations:** 1 Cognitive Brain Research Unit, Department of Psychology and Logopedics, Faculty of Medicine, University of Helsinki, Helsinki, Finland; 2 Centre of Excellence in Music, Mind, Body and Brain, University of Helsinki, Helsinki, Finland; 3 Queensland Aphasia Research Centre, Brisbane, QLD Australia; 4 School of Health and Rehabilitation Sciences, The University of Queensland, Brisbane, QLD Australia; 5 Centre of Research Excellence in Aphasia Recovery and Rehabilitation, La Trobe University, Melbourne, VIC Australia; 6 BABA Center, Pediatric Research Center, Department of Clinical Neurophysiology, Children’s Hospital, Helsinki University Hospital and University of Helsinki, Helsinki, Finland; 7 Department of Neurology, Helsinki University Hospital and Department of Neurosciences, University of Helsinki, Helsinki, Finland

**Keywords:** Language, Stroke

## Abstract

Theories expounding the neural relationship between speech and singing range from sharing neural circuitry, to relying on opposite hemispheres. Yet, hodological studies exploring their shared and distinct neural networks remain scarce. In this study, we combine a white matter connectometry approach together with comprehensive and naturalistic appraisal of verbal expression during spoken language production and singing in a sample of individuals with post-stroke aphasia. Our results reveal that both spoken language production and singing are mainly supported by the left hemisphere language network and projection pathways. However, while spoken language production mostly engaged dorsal and ventral streams of speech processing, singing was associated primarily with the left ventral stream. These findings provide evidence that speech and singing share core neuronal circuitry within the left hemisphere, while distinct ventral stream contributions explain frequently observed dissociations in aphasia. Moreover, the results suggest prerequisite biomarkers for successful singing-based therapeutic interventions.

## Abstract (web summary)

Analysis of diffusion MRI and word production through spoken language and singing in a cohort of 45 stroke survivors demonstrates that impaired speech and singing ability relies on the disruption of partially overlapping brain circuits.

## Introduction

The prevailing framework of the functional neuroanatomy of speech processing suggests that it is principally organized around two perisylvian streams: a ventral stream connecting the middle and inferior temporal regions and ventrolateral prefrontal cortex through the extreme capsule via inferior fronto-occipital fasciculus, involved in mapping sound onto meaning; and a dorsal stream, connecting the superior temporal and premotor regions through the arcuate and superior longitudinal fascicle, engaged in mapping sound onto articulatory-based representations^1–5^. Both streams are considered left-dominant, although the ventral stream is more bilaterally organised^3^. This framework for the neural organization of speech processing has been extrapolated to language more generally, largely through neuroimaging studies in patients with aphasia, an acquired language impairment most commonly resulting from stroke, showing that deficits in semantic processing have been associated with lesions in the ventral stream, whereas impairments in sensory-motor integration have been linked to lesions in the dorsal stream^6–8^.

Interestingly, the extent of damage to the left arcuate fasciculus, a major dorsal white matter pathway, historically associated with repetition tasks, has most commonly been linked to both long-term spoken language production outcomes in aphasia^9^, and anomia, the most common residual language deficit in aphasia^10^. In aphasia research, relatively simple speech production tasks, such as single word naming tasks and short repetition tasks, are frequently used, instead of more comprehensive assessment of language such as connected speech production, or propositional language. Moreover, most neuroimaging studies on the neural organization of speech and spoken language production have used indirect measures to quantify and identify white matter tracts (such as lesion load^11^). Whilst there is growing interest in exploring language in contexts that are closer to everyday communication, such as samples of connected spoken language where subjects produce a continuous sequence of spontaneous speech, for example, in response to a complex picture or a broad question. Studies applying such measures with advanced hodological methods are scarce.

Singing constitutes another channel for vocally producing verbal material that naturally increases connectedness between syllables and words and, in this respect, resembles connected spoken language production. Dating back to Darwin, evolutionary models of language have posited the existence of a musical protolanguage, a singing-like emotionally grounded vocal communication system, as an early precursor in the development of spoken language^12^, suggesting that the human brain may have evolved and organized in parallel to support both speech and singing. Modern theoretical neuroanatomical models have espoused that similar to speech, singing also entails left dorsal and ventral streams for the use and integration of auditory and motor systems^13,14^. Functional neuroimaging studies in healthy subjects have lent support to this view, showing a considerable overlap in the neural networks related to speech and singing, but also suggesting that singing engages frontotemporal areas more bilaterally^15,16^. Evidence for the structural neuroanatomy of singing is, however, limited, especially regarding which brain regions and pathways are causally linked to singing production.

Nonfluent aphasia provides a notable approach to the similarities and differences of neural networks supporting speech and singing. Case descriptions dating back to the 18th century recount that people with severe deficits in spoken language production may have preserved ability to produce the lyrics and the melody of familiar songs through singing^17,18^. Although such anecdotal evidence has laid the foundation for singing-based rehabilitation methods formally used in aphasia rehabilitation since 1970’s^19–21^, the neural correlates of preserved singing ability in aphasia are insufficiently understood and with no known diffusion imaging studies on the subject. Based on the findings emphasizing the bilaterality of vocal music processing and case reports and studies in people with aphasia, it has been hypothesized that singing recruits contralateral right hemisphere regions that are able to compensate for language deficits, especially following a more extensive stroke^19,22^.

Acknowledging that disruptions in speech and spoken language production can coincide with deficits in producing words through singing^23–25^, it is conceivable that these tasks engage anatomically overlapping networks, at least to some degree. In our recent study^26^ utilizing voxel-based morphometry (VBM) and lesion-symptom mapping (VLSM) in patients with chronic post-stroke aphasia, we found that impairments in connected spoken language production and repetition, as well as sung repetition, were associated with damage to left frontal, temporal, and parietal structures. Impairments in singing the words of a familiar song, in turn, were associated with damage only to the left temporal regions, namely middle and superior temporal gyri and middle and superior temporal poles. These findings not only challenge the traditional view of the neural architecture subserving the potentially preserved singing ability in nonfluent aphasia, but also indicate that spoken language production and singing rely partly on the same neural networks. Their hodological organization in the human brain, however, have remained uncharted.

To our knowledge, this is the first study to utilize comprehensive and naturalistic appraisal of participants’ verbal expression^11,27^ during spoken language production and singing in combination with a diffusion MRI analytic approach to map the shared and distinct structural white matter connectomes subserving both spoken language production and singing. Specifically, we assessed 45 participants with chronic post-stroke aphasia and their efficacy of both connected spoken language production and singing as well as efficacy of repeating identical phrases in spoken and sung format. We acquired multi-shell diffusion MRI data and employed quantitative anisotropy (QA)^28^ based connectometry that uses permutation testing to label white matter tracts associated with variables of interest. This approach has recently been applied to quantifying white matter tracts associated with word production^29^ and verb retrieval^30^ in aphasia. QA-based connectometry has proven superior to traditional track- and voxel-based analyses applying fractional anisotropy (FA)^28,31^. Based on our previous results^26^ and neuroanatomical models of speech^1–5^ and singing^13,14^, we hypothesized that (1) the structural network supporting connected spoken language production efficacy in chronic post-stroke aphasia is centred upon the left dorsal stream, whereas connected singing efficacy is related to preserved left ventral stream as well as right homologous language network; (2) the core left structural network supporting singing in aphasia is, at least partly, independent of spoken language production efficacy, but the extent to which the additional networks are linked to singing is mediated by the severity of spoken language production deficit; and (3) both spoken and sung repetition efficacy are associated with the left dorsal stream, with the latter also engaging right hemisphere tracts.

## Results

### Behavioural associations of spoken language and singing measures

To quantify the behavioural relationship of speech and singing in the domains of production efficacy and repetition, two simple linear regression analyses were calculated evaluating the relationships of spoken language production and connected singing efficacy as well as spoken and sung repetition efficacy. In the production efficacy model including connected singing efficacy as a dependent variable and spoken language production as a predictor, it was found that spoken language production significantly predicted connected singing efficacy (β = 0.359, *p* = 0.018), explaining 13% of the variance. The second model (repetition) included sung repetition efficacy as a dependent variable and spoken repetition efficacy as a predictor and showed that spoken repetition efficacy significantly predicted sung repetition efficacy (β = 0.884, *p* < 0.001), explaining 78% of the variance.

### Networks supporting connected spoken language production and connected singing efficacy

**Fig. 1:**
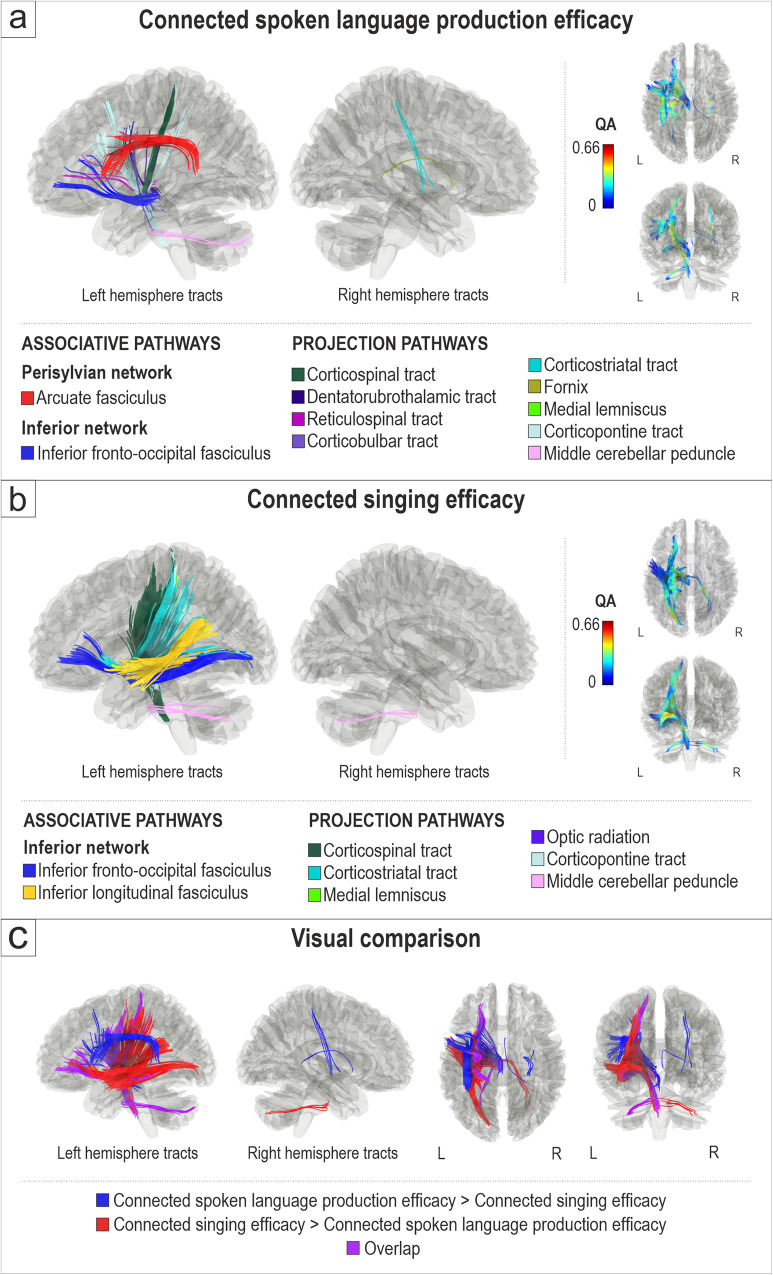
White matter connectomes subserving connected spoken language production efficacy and connected singing efficacy. Structural pathways positively associated (FDR < 0.0083) with (**a**) connected spoken language production efficacy (*n* = 45) and (**b**) connected singing efficacy (*n* = 43). **c** Visual comparison of tracts associated specifically with connected spoken language production efficacy (spoken language production > singing; blue), with connected singing efficacy (singing > spoken language production; red) and their overlap (purple). L = left, R = right.

First, we evaluated the white matter connectomes related to connected spoken language production efficacy (*n* = 45) and connected singing efficacy (*n* = 43) in participants with aphasia (Fig. 1a–c). Better connected spoken language production efficacy, as measured by mean correct information units per minute in three spontaneous spoken language production tasks, was associated with greater QA in the left dorsal stream (arcuate fasciculus), left ventral stream (inferior fronto-occipital fasciculus), and projection pathways in the left (corticospinal tract, corticopontine tract, dentatorubrothalamic tract, reticulospinal tract, corticobulbar tract, medial lemniscus, middle cerebellar peduncle) and right (corticostriatal tract, fornix) hemisphere (FDR < 0.0083; Fig. 1a). There were no negative associations between connected spoken language production efficacy and QA.

Better connected singing efficacy, as measured by correct words sung per minute (of a well-known song), was associated with greater QA in the left ventral stream (inferior fronto-occipital fasciculus, inferior longitudinal fasciculus) and projection pathways in the left (corticospinal tract, corticostriatal tract, corticopontine tract, medial lemniscus, optic radiation, middle cerebellar peduncle) and right (middle cerebellar peduncle) hemisphere (FDR < 0.0083; Fig. 1b). There were no negative associations between connected singing and QA.

The visual comparison of white matter connectivity positively associated with connected spoken language production efficacy and connected singing efficacy (Fig. 1c) shows a degree of (i) shared connectivity in the anterior part of the left ventral stream between spoken language and singing and (ii) distinct (non-shared) connectivity which was stronger in the left dorsal stream and left anterior projection pathways for spoken language and (iii) in the posterior part of the left ventral stream and left posterior projection pathways for singing.

To control for the effects of comorbid speech motor difficulties, connectometry analyses for connected spoken language production efficacy and connected singing efficacy were reproduced with Boston Diagnostic Aphasia Examination (BDAE)^32^ articulatory agility score as a third covariate. The abovementioned reported results remained largely unaffected when controlling for the impairments in motor articulatory praxis (Supplementary Fig. 1). Furthermore, we carried out additional analyses controlling for the effects of (i) time post stroke, (ii) choir singing experience in years, and (iii) Montreal Battery of Evaluation of Amusia (MBEA) score on connected spoken language production efficacy and connected singing efficacy. The main results remained largely the same (Supplementary Fig. 1).

### Networks supporting connected singing in relation to the severity of spoken language deficits

In order to determine whether the severity of the spoken language production deficit mediated the white matter connectome related to connected singing efficacy in aphasia, a median split of the connected spoken language production efficacy score was used to divide the participants to those with less severe (*n* = 22, mean = 52.0, SD = 16.4) and more severe (*n* = 21, mean = 9.4, SD = 12.0) deficits in connected spoken language production efficacy.

**Fig. 2:**
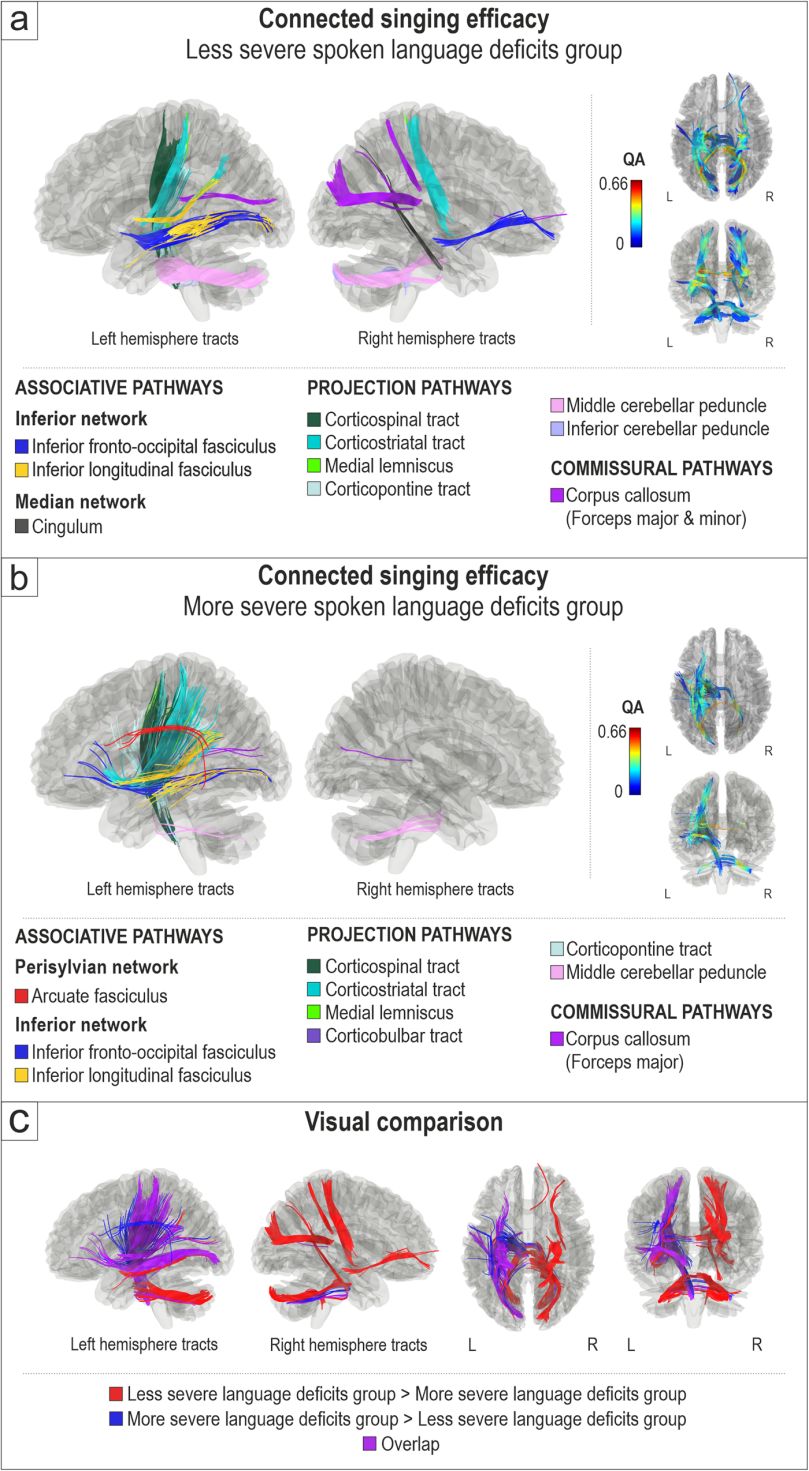
White matter connectomes subserving connected singing efficacy in relation to spoken language deficits. Structural pathways positively associated (FDR < 0.0083) with connected singing efficacy in (**a**) less severe spoken language deficits group (*n* = 22) and (**b**) more severe spoken language deficits group (*n* = 21). **c** Visual comparison of tracts associated specifically with less severe language deficits (less severe deficits group > more severe deficits group; red), with more severe language deficits (more severe deficits group > less severe deficits group; blue) and their overlap (purple). L = left, R = right.

In the less severe group, better connected singing efficacy was associated with greater QA in the left (inferior fronto-occipital fasciculus, inferior longitudinal fasciculus) and right (inferior fronto-occipital fasciculus) ventral stream, left (corticospinal tract, corticostriatal tract, corticopontine tract, medial lemniscus, inferior and middle cerebellar peduncle) and right (corticostriatal tract, inferior and middle cerebellar peduncle) projection pathways, right median network (cingulum), and commissural pathways (corpus callosum, forceps major, forceps minor) (FDR < 0.0083; Fig. 2a). In this group, no negative associations between connected singing efficacy and white matter connectivity were found.

In the more severe group, better connected singing efficacy was associated with greater QA in the left ventral stream (inferior longitudinal fasciculus, inferior fronto-occipital fasciculus), left dorsal stream (arcuate fasciculus), left projection pathways (corticospinal tract, corticostriatal tract, medial lemniscus, corticopontine tract, corticobulbar tract, middle cerebellar peduncle), right projection pathways (middle cerebellar peduncle) and commissural pathways (corpus callosum, forceps major) (FDR < 0.0083; Fig. 2b). Additionally, in this group, poorer connected singing efficacy was associated with greater QA in the right dorsal stream (superior longitudinal fasciculus) and right projection pathways (corticospinal tract) (FDR < 0.0083; Supplementary Fig. 2).

The visual comparison of white matter connectivity positively associated with connected singing efficacy in the less and more severe groups (Fig. 2c) shows that connected singing efficacy was linked to (i) the middle/posterior part of the left ventral stream and the left projection pathways in both less and more severe groups and, additionally, (ii) the left dorsal stream and the anterior part of the left ventral stream in the more severe group and (iii) the right ventral stream, cingulum, and projection pathways in the less severe group.

### Networks supporting spoken and sung repetition efficacy

**Fig. 3:**
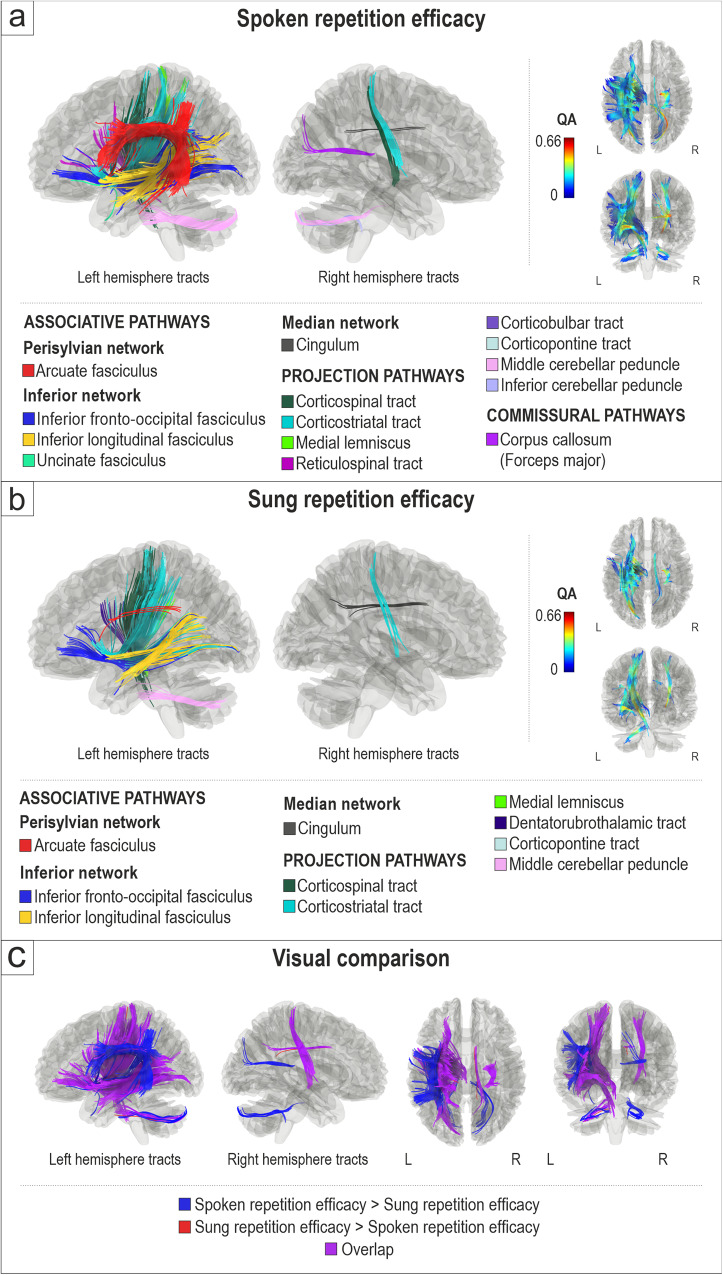
White matter connectomes subserving spoken and sung repetition efficacy. Structural pathways positively associated (FDR < 0.0083) with (**a**) spoken repetition efficacy (*n* = 45) and (**b**) sung repetition efficacy (*n* = 45). **c** Visual comparison of tracts associated specifically with spoken repetition efficacy (spoken repetition > sung repetition; blue), with sung repetition efficacy (sung repetition > spoken repetition; red) and their overlap (purple). L = left, R = right.

To control if the pattern of shared and distinct structural networks between connected spoken language and singing in aphasia also emerged in more basic auditory-motor verbal tasks, we also analysed the white matter connectomes related to the repetition of short spoken and sung phrases. Better spoken repetition efficacy (correct words per minute) was associated with greater QA in the left dorsal stream (arcuate fasciculus), left ventral stream (inferior fronto-occipital fasciculus, inferior longitudinal fasciculus, uncinate fasciculus), left (corticospinal tract, corticostriatal tract, corticopontine tract, reticulospinal tract, corticobulbar tract, middle cerebellar peduncle, medial lemniscus) and right (corticospinal and corticostriatal tracts, middle and inferior cerebellar peduncle) projection pathways, right median network (cingulum) as well as commissural pathways (corpus callosum, forceps major) (FDR < 0.0083; Fig. 3a). Negative associations between spoken repetition efficacy and white matter connectomes were not found.

Better sung repetition efficacy (correct words per minute) was associated with greater QA in the left dorsal stream (arcuate fasciculus), left ventral stream (inferior longitudinal fasciculus, inferior fronto-occipital fasciculus), left (corticospinal tract, corticostriatal tract, corticopontine tract, dentatorubrothalamic tract, middle cerebellar peduncle, medial lemniscus) and right (corticostriatal tract) projection pathways, and right median network (cingulum) (FDR < 0.0083; Fig. 3b). Negative correlations between sung repetition efficacy and white matter connectomes were not found.

The visual comparison of white matter connectivity positively associated with spoken and sung repetition efficacy (Fig. 3c) shows that there was a strong overlap between spoken and sung repetition in the left ventral stream and in the bilateral projection pathways, but the left dorsal stream had a stronger link to spoken repetition than to sung repetition.

### *Post hoc* analysis

To further disentangle the structural networks that support producing words through singing in severe aphasia, we also conducted a *post hoc* subgroup analysis comparing the white matter connectomes between participants with severe aphasia who were unable to produce any words through singing (*n* = 5, connected spoken language efficacy *M* = 0, range 0; connected singing efficacy *M* = 0, range 0) and participants with severe aphasia who showed at least partially preserved ability to produce words through singing (*n* = 6, connected spoken language efficacy *M* = 0.4, range 0–1.5; connected singing efficacy *M* = 30.9, range 18.7–63.9). These groups did not show significant differences in age (*p* = 0.416), time from stroke (*p* = 0.938), lesion volume (*p* = 0.228) or BDAE articulatory agility score (*p* = 0.182).

**Fig. 4:**
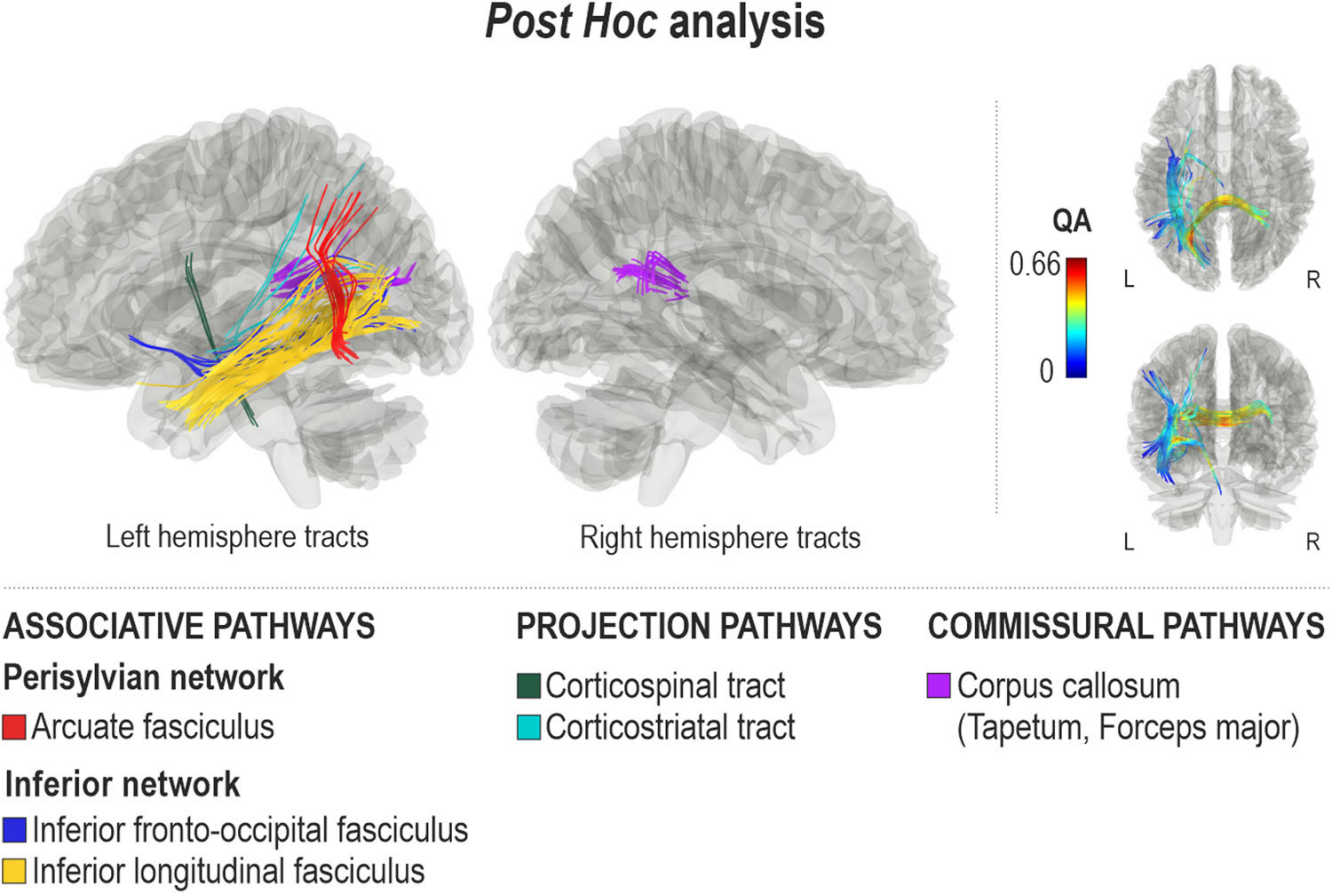
White matter connectomes subserving preserved connected singing abilities in patients with severe connected spoken language production deficits. Comparison (B > A) of subgroups with severe spoken language production impairment and either inability (subgroup A, *n* = 5) or preserved ability (subgroup B, *n* = 6) to produce words through singing (FDR = 0.001).

Compared to the participants with inability to produce words through both speech and singing, the participants with inability to produce words through speech yet relatively preserved ability to produce words through singing showed higher QA in the left ventral stream (inferior longitudinal fasciculus, inferior fronto-occipital fasciculus), left dorsal stream (arcuate fasciculus), left projection pathways (corticospinal tract, corticostriatal tract), and commissural pathways (corpus callosum, tapetum and forceps major) (FDR = 0.001) (Fig. 4). Higher QA values in opposite direction were not observed (FDR = 1).

## Discussion

Using state-of-the-art white matter connectometry analysis, this study has identified shared and distinct structural white matter connectomes subserving both spoken language production and singing in aphasia. Our findings indicate that both connected spoken language production and singing are mainly supported by the left hemisphere language network and general projection pathways. However, whereas connected spoken language production efficacy relied on both left dorsal and ventral stream pathways, adhering to dual-pathway models of speech processing^3–5^, connected singing efficacy was associated primarily with the left ventral stream. The present study offers a feasible hodological explanation for the spared ability to produce words through singing in aphasia and why impairments in speech production can coincide with those in singing. Strikingly, the results provide a counterargument for the role of right hemisphere structures in singing words in aphasia, which, in fact, seems to rely on the intactness of the ventral stream within the left perisylvian language network as well as projection pathways.

Addressing the limitations of the classical neurological models of language, modern models of speech and language processing have proceeded from modulatory views to emphasize the relevance of neuroanatomical networks and subcortical structures^3–5^. A wealth of lesion-mapping studies associating damage to the dorsal and ventral streams with aphasia^7,9,11,33,34^ have lent support to a dual-stream model of language, but hodological studies based on modern diffusion MRI methods remain scarce. To our knowledge, this is the first study to use a whole-brain hodological approach to map the white matter pathways involved in connected spoken language production. A composite score of three different discourse genres was used to attain a valid estimate of participant’s connected spoken language production efficacy, given that in aphasia test-retest reliability is poor when using single short speech samples^27^ and there is considerable within-subject variability in performance across discourse genres^11^. Using an outcome measure comprised of multiple discourse genres is of great relevance for post-stroke aphasia^35^, in which the lexical richness, coherence and completeness of discourse are reduced at varying levels. Given that discourse-level speech production is a complex skill, including processes of conceptualization, formulation, and articulation^36^, it combines not only motor-phonological and lexical-semantic processing, but also executive processing^11,34^ including auditory feedback perception and control^37^. This complexity is reflected in the present results, which show that (i) discourse-level spoken language production necessitates the interaction of both dorsal and ventral streams, providing further evidence for the dual-stream model, and moreover, (ii) point to a wider network involving projection pathways, including corticospinal, corticostriatal, corticopontine, and cerebellar tracts, that are typically linked to cognitive control^38^ and motor functions^30^.

Paralleling the spoken language efficacy assessment, patients’ efficacy in singing words of a well-known song was evaluated to allow comparisons to spoken language as well as its universal replication as an outcome measure. The theoretical models of singing suggest that the left dual-stream language system supports the organization of singing in the use and integration of auditory and motor systems^13,14^. Functional neuroimaging studies in healthy subjects have shown that frontotemporal structures are more bilaterally engaged during singing compared to speech^15,16^. More recently, we have shown that lesions in the left temporal regions, mainly ventrally, were associated with deficits in singing words of a familiar song^26^. For these reasons, we anticipated that connected singing efficacy would be associated with the left ventral stream as well as the right hemisphere homologous language network. Our results partially confirmed this hypothesis by showing that connected singing efficacy was indeed associated with the left ventral stream in addition to left-lateralized domain-general projection pathways, paralleling the results from the spoken language production analysis. Compared to connected spoken language production, an important and highly interesting distinction was that we did not find an association between the left dorsal stream and connected singing efficacy in the main analysis. The distinction between connected spoken language production and connected singing efficacy was further underlined when their behavioural association was examined. While significantly associated, connected spoken language production efficacy explained only 13% of connected singing efficacy variance. As observed in the results, this suggests that they rely on similar structural circuitry, but also highlight their different emphasis on ventral and dorsal pathways. In turn, spoken repetition efficacy explained 78% of sung repetition efficacy variance arguing for strong correlation between these two measures. Structurally, the left ventral stream was associated with both spoken and sung repetition, whereas the left dorsal stream showed a much stronger link to spoken than sung repetition. Together, these observations most likely reflect that both spoken and sung repetition tasks, comprising of repetition of multiple words, is highly reliant on higher-level language comprehension mediated by the ventral stream, rather than sublexical repetition subserved by the dorsal stream. With respect to the dual-stream system, these converging findings indicate that the sung production of words in aphasia relies primarily on the integrity of the left ventral stream, both at the basic auditory-motor level (repetition) and in more naturalistic (connected) singing.

Within the left ventral stream, another interesting difference between connected spoken language production and connected singing was that while they showed shared connectivity in the anterior part of the stream, the posterior part, which comprised the inferior longitudinal fasciculus and the posterior half of the inferior fronto-occipital fasciculus, was linked only to singing. Also, in the subgroup analyses of patients with less/more severe deficits in spoken language production efficacy, connected singing was linked to the left inferior fronto-occipital fasciculus, running from the posterior temporal and parieto-occipital cortex to the ventrolateral frontal cortex, in both subgroups. Similarly, our *post hoc* analysis suggested that within the severe aphasia subgroup those patients who had relatively preserved ability to produce words through singing had higher connectivity in the left ventral stream as well as in the left posterior dorsal stream connecting posterior temporal and parietal regions and the posterior corpus callosum (tapetum) connecting the left and right temporal lobes compared to patients who were unable to produce words through singing. Notably, these three pathways converge in the left posterior temporal region, which we previously found to be structurally linked to the sung production of words^26^. Together, these findings suggest that the preserved ability to produce words through singing in severe aphasia is underpinned by spared structural connectivity especially between the left posterior temporal cortex and the left ventrolateral frontal cortex via the long ventral tract, which provides access to lexical-semantic and conceptual information^2^ and enables the recall of the familiar song lyrics^39^. Additional important hubs might include the left parietal cortex via the posterior dorsal tract, which provides access to phonological information and to the sensorimotor interface^3^ and the right superior temporal cortex via the tapetum, which provides access to spectral/melodic features of the song^40,41^.

Previous studies have postulated that patients with persistent connected spoken language production deficits may benefit from speech entrainment, denoting a utilization of audio-visual feedback for real-time mimicking of fluent speech^42^. Interestingly, the gain of assisted speech was found in patients with damage to the anterior dorsal stream, but relatively preserved left ventral stream structures, including the posterior middle temporal gyrus and the inferior fronto-occipital fasciculus, suggesting that speech entrainment may act as a conduit for the ventral stream to engage lexicon and conceptual encoding^33^. Singing the lyrics of an over-learnt song, for which a person has previously established an internal auditory-motor model, may instantiate a type of vocal expression that resembles entrained speech, thus providing a scaffold for verbal expression that makes use of semantic cues for word production mediated by the ventral stream and places less demand on sensory-motor mapping of sound to articulation mediated by the dorsal stream. Relying on the spared ventral stream could also explain why the repetition and completion of familiar song lyrics can be more efficient in nonfluent aphasic patients when presented in sung format (as opposed to spoken format)^43,44^ and why they can also learn novel lyrics better when singing along an auditory model^45^. Thus, the engagement of the spared ventral stream not only provides a plausible hodological explanation for the preservation of singing production ability in aphasia but may provide an important avenue for therapeutic interventions.

Contrary to our hypotheses, no clear associations between singing efficacy and right hemisphere pathways were detected. This was further underlined in our explorations of split subgroups revealing that irrespective of the severity of the spoken language production deficit, connected singing was associated with left ventral and projection pathways and commissural pathways. Only participants with less severe spoken language production deficits showed additional right ventral, medial, and projection pathways as part of the connectome for connected singing. This finding may possibly reflect the supplementary engagement of the right hemisphere auditory-motor system to facilitate the melodic processing of the song, which is known to be closely linked to lexical/phonological processing in the healthy brain^46^ and to lyrical production also in aphasia^44^. Overall, this pattern of results is surprising given that the role of the right hemisphere structures in singing, especially in more severe aphasia, has been emphasized for over five decades^19^. Whereas the searchlight has focused on finding treatment-associated tract changes only in the right hemisphere in patients with nonfluent aphasia undergoing singing-based therapy (i.e., melodic intonation therapy)^47–49^, left hemisphere pathways have remained unexplored. Moreover, this evidence has derived from several small case series, leaving the hodological correlates of singing-based therapy in aphasia largely uncharted. However, it is feasible that in patients with very large lesions occupying both left dorsal and ventral streams, the treatment-related neuroplasticity takes place in contralesional intact hemisphere. Yet, our results suggest that the gateway for speaking through singing in aphasia may lie within the left hemisphere language network and domain-general pathways. Future studies determining the prerequisite biomarkers for successful singing-based therapies in aphasia would provide broader predictive value and validity of the current results, could aid patient selection for music-based therapies and allow clinicians to reliably predict outcomes on a single-subject level in a clinical setting.

Our exploration of connected singing was limited to producing words of a familiar song, as it represents the foremost instance of preserved singing ability in aphasia and constitutes a universally replicable outcome measure. Moreover, singing an over-learnt song does not rely on reading lyrics, an important point given that reading is often impaired in aphasia. Evaluation of connected singing efficacy also enabled us to reasonably compare its structural connectome to that of spoken language production efficacy. Both tasks used in the study mitigate the confound of working memory on language production, allowing us to focus on the production of language (main outcome in the study). However, it must be borne in mind that the tasks are not identical in the two domains (speech, singing) and a fully comparable singing-based task to the connected spoken language production task would translate coarsely to improvising a novel song which would then necessitate improvising both the lyrics and the melody and would require additional neural resources, tapping into additional interhemispheric as well as right-hemispheric networks involved in acoustic information as well as musical syntactic processing^50^. For these reasons, we did not directly and quantitatively compare the two tasks. Rather than the singing performance itself, the pivot of our exploration was the neuroanatomy of spared sung word production in aphasia, a classical observation in neurology and neuroscience^17^. Singing is a multidomain function that, beyond producing words, involves sensory-motor control to achieve correct pitch and rhythm as well as conveying emotions. Therefore, future studies are required to elucidate the neural networks subserving successful melodic and rhythmic production of a song as well as the networks related to singing of a novel song. These circuits may extend beyond the now observed pattern of results to include, not only learning-related structures, but also right hemisphere structures that have been linked to pitch-matching accuracy during singing in both healthy subjects^51^ and after stroke^52^ as well as deficits in processing rhythmic-melodic acoustic information in both language and musical domains^53^. Future studies including patients with both left and right hemisphere lesions as well as an age-matched healthy control group are needed to provide external validity for the current pattern of results on singing and further probe the multidomain functions, including melodic-rhythmic abilities related to singing. Moreover, owing to the relatively small sample sizes in the analyses evaluating the local connectome associations with connected singing efficacy in patients with less (*n* = 22) and more severely (*n* = 21) impaired connected spoken language production as well as in the post hoc analysis (*n* = 11), these results should be interpreted with caution. For example, the only negative associations between QA and singing efficacy as well as only positive associations between the right hemisphere and singing efficacy were observed in these analyses, raising the possibility of false positive findings in these suboptimally powered analysis. While we have utilised stringent statistical thresholds to avoid false positives in our findings, future large-scale studies are needed to confirm these results.

To conclude, in addition to presenting unique inherent human proclivities and relying on the same anatomical vocal tracts, speech and singing are underpinned by the same neuroanatomical networks, leaning on left-lateralized language network as well as general projection pathways. However, whilst spoken language production relied on the left dual-stream system, word production through singing was primarily associated with the left ventral stream. From the perspective of evolutionary neuroscience, these results imply at least partial parallel development of speech and singing, complying with a conservation principle that the mammalian brain optimally develops using minimum expenditure of wiring costs and optimal conductivity speed^54^. Our results also provide a feasible hodological explanation for the classical observation in neurology of spared ability to produce words through singing in aphasia as well as suggesting that the gateway for singing production may lie within the left hemisphere language network and domain-general pathways. Moreover, the results suggest prerequisite biomarkers for successful singing-based therapies in aphasia.

## Methods

### Participants

Forty-five participants (25 females, mean age = 64.4, SD = 10.2) with post-stroke aphasia were recruited from Helsinki and Turku regions during 2017–2019 through patient organizations (Helsinki-Uusimaa and Turku region Stroke Association, Finnish Brain Association) and clinical speech therapists. All participants had chronic aphasia (>6 months since onset) due to left hemisphere stroke, confirmed by clinical assessment, were over 18 years and spoke Finnish as a native language and had no hearing deficits, severe cognitive impairments (affecting their ability to co-operate and provide an informed consent), substance abuse or neurological or psychiatric comorbidities. A sample of 45 participants should provide power of at least 0.8 to detect relationships between language skills and white matter connectivity patterns as suggested by previously reported data on white matter connectivity in post-stroke aphasia^29^. Demographic and clinical information of the participants is presented in Table 1. The study was conducted in accordance with the Declaration of Helsinki and approved by the Ethics Committees of the Hospital Districts of Helsinki-Uusimaa and Southwest Finland. Informed consent was obtained from all recruited participants.

**Table 1:** Demographic and clinical characteristics of the participants (*N* = 45).

Demographical	
Age (years)	64.4 (10.2: 49.0)
Sex (female/male)	25/20
Handedness (right/left/both)	39/5/1
Education level^a^	3.0 (1.4: 5.0)
Music background	
MBEA (correct %)	70.9 (13.1: 50.0)
Singing lessons (years)	0.5 (2.7: 18.0)
Choir singing (years)	3.6 (9.4: 34.0)
Clinical background	
Time from stroke (years)	9.0 (7.7: 37.8)
Lesion volume (cm^3^)	95.8 (87.6: 320)
BDAE severity rating	3.0 (1.4: 4.0)
WAB Aphasia Quotient	63.4 (34.1: 95.1)
WAB Spontaneous Speech	12.9 (7.1: 20)
WAB Naming	6.2 (3.6: 9.9)
WAB Repetition	6.5 (3.5: 10.0)
Speech and spoken language production outcomes	
Connected spoken language production efficacy^b^	30.8 (25.5: 106.0)
Spoken repetition efficacy^c^	46.0 (27.1: 86.0)
BDAE verbal agility	7.2 (4.6: 14.0)
Singing outcomes	
Connected singing efficacy^c^	42.0 (28.8: 113.5)
Sung repetition efficacy^c^	23.9 (14.5: 56.2)

Data are mean (SD: range) unless otherwise reported.

*BDAE* Boston Diagnostic Aphasia Examination, *MBEA* Montreal Battery of Evaluation of Amusia, *WAB* Western Aphasia Battery.

^a^Education level according to the Unesco International Standard Classification of Education: range 1 (primary education) to 6 (doctoral or equivalent level).

^b^Correct information units per minute.

^c^Correct words per minute.

### Behavioural assessments and procedure

Behavioural tasks were completed as a part of a baseline neuropsychological assessment (taking approximately 2 h) of a randomized controlled clinical trial and were administered by a trained psychologist. To attain clinical information about the aphasia severity and overall language impairment of the participants, BDAE^32^ severity rating and Western Aphasia Battery (WAB)^55^ Aphasia Quotient were determined. Initial aphasia severity rating of each participant was evaluated by the recruiting psychologist based on the recruitment interview and the final rating was validated based on observations during the behavioural assessment. To obtain an estimate of the level of motor speech difficulties in our sample, the BDAE articulatory agility task was also included in the behavioural assessment battery.

Spoken and sung production tasks were digitally recorded with Presentation software (Neurobehavioral Systems, Inc., Berkeley, CA, www.neurobs.com) and later transcribed for scoring that was conducted by a licensed neuropsychologist (S-T.S). PRAAT software (www.praat.org) was used for segmenting and labelling the recordings of connected spoken language and singing production and spoken and sung repetition tasks and for calculating the total production time of each task. The production time was calculated from the onset to the end of production, excluding statements and questions that were clearly unrelated to the task.

Connected spoken language production efficacy was evaluated with a composite score of production rate in three different tasks: (1) WAB Picture Description task (“Picnic”)^55^, (2) Sequential picture description (“Argument”)^56^ and (3) Personal information (responding to a question “Tell me what you usually do on Sundays”)^56^. The participants were encouraged to continue spoken language production for at least 1 min (60 s) in each task and there was no upper limit for the production time. The participants were not provided with hints or further questions to prompt production.

To assess the communicative informativeness and efficacy of connected spoken language production, the number of correct information units^56^ were calculated on each task for each participant. Minor phonemic paraphasia was admitted if the content was clearly recognizable. A composite score of mean correct information units per minute was derived from the three discourse samples to evaluate spoken language production efficacy. There was no missing data.

Over-learnt singing (here, connected singing) was assessed through spontaneously singing a generally familiar song, Jaakko-kulta (Finnish version of *Frere Jacques/Brother John*) two times consecutively. To provide the production rate of sung words, the number of correct words and the length of the production (starting from the onset of production) were calculated. For a comparability with spoken language production efficacy, minor distortions were accepted as correct units. The digital recordings of two participants were lost due to technical malfunction.

The participants also completed a repetition task in which they were asked to repeat 16 pre-recorded phrases that were presented through headphones. On the first round, the stimuli were presented with normal prosody (spoken format) and on the second round, with melodic intoning (sung format). The phrases were identical on both rounds and varied from 1 to 5 words (in total 42 words). The audio models were pre-recorded by a music therapist and the production of the sung phrases followed the principles of melodic intonation therapy^19^. For spoken phrases, the length of the audio stimuli varied from 1.07 to 3.95 s and for the sung stimuli from 2.02 to 7.90 s. The first repetition attempt of each phrase was used for scoring. The number of correct words (consistently very minor phonemic paraphasia accepted) were determined and correct words per minute were calculated to yield production rate and attain comparability between all outcome measures. There was no missing data.

### MRI data acquisition

Participants were scanned on a 3-Tesla Siemens Skyra scanner at the Helsinki and Uusimaa Hospital District Medical Imaging Centre or at the Medical Imaging Centre of Southwest Finland. Multi-shell diffusion-weighted MRI (DW-MRI) with 13 non-diffusion weighted volumes and 130 diffusion weighted volumes (30 volumes with b = 1000 s/mm^2^ and 100 volumes with b = 2500 s/mm^2^) were obtained with the following parameters: repetition time (TR) = 5000 ms, echo time (TE) = 104 ms, field of view (FOV) = 240 × 240 mm, voxel size 2.0 × 2.0 × 2.0 mm^3^.

### DW-MRI data preprocessing and reconstruction

The DW-MRI data were denoised using Marchenko–Pastur PCA method^57^ implemented in the MRTrix3 software^58^. Next, Gibbs ringing artefact correction was performed with a method based on local subvoxel-shifts^59^. After this, the DW-MRI data were reconstructed in the Montreal Neurological Institute (MNI) space in DSI Studio (http://dsi-studio.labsolver.org) using q-space diffeomorphic reconstruction (QSDR)^60^ with a 1.25 diffusion sampling length ratio, permitting the construction of spin distribution functions^61^ (SDFs) in spatially normalized diffusion data (see Supplementary Fig. 3). The b-table orientation was monitored with an automatic quality control routine using the fibre coherence index^62^. The R^2^ values indicating the goodness-of-fit between template and anisotropy map were inspected for each participant. The spatial output resolution of SDF maps was 2 mm isotropic. To verify normalization quality, forceps major and minor were used as an anatomical benchmark and manually explored^29^. Restricted Diffusion Imaging (RDI) was used to quantify restricted diffusion^63^. Derived from the peak orientations on SDF, QA was extracted as the local connectome fingerprint^64^ to be used in the connectometry analysis.

### Reconstruction of lesions

For each patient, a high-resolution T1-weighted image was obtained (TR = 1800 ms, TE = 2.27 ms, voxel size = 1.0 × 0.98 × 0.98 mm^3^, matrix = 256 × 256). First, individual T1 images were reoriented according to the anterior commissure. Then, binary masks of the stroke lesions were created using MRIcron software^65^ by manually depicting the precise lesion boundaries on a slice-by-slice basis using T1 images of individual patients. A sum image of all patients' lesions is shown in Supplementary Fig. 4. T1 images and lesions masks were then preprocessed using the Statistical Parametric Mapping software (SPM12, www.fil.ion.ucl.ac.uk/spm/) under MATLAB R2018b and segmented using Unified Segmentation^66^ with medium regularization and SPM12 IXI data set tissue probability maps. Patient-specific lesion masks were used as cost function masking to prevent post-registration lesion shrinkage and out-of-brain distortion^67^ and to achieve optimal normalization of MRI images containing stroke lesions^68^. Damaged voxels were masked out to achieve an accurate segmentation and spatial normalization. The segmented tissue probability maps were then modulated and normalized to Montreal Neurological Institute (MNI) space, together with the lesion masks. Lastly, residual inter-individual variability was reduced by smoothing the tissue probability maps and lesions masks using an isotropic spatial filter (FWHM = 6 mm).

### Statistics and reproducibility

DW-MRI connectometry^31^ analyses were performed with DSI Studio. Connectometry is a relatively new statistical method that includes mapping and analysis of the degree of connectivity between adjacent voxels within a white matter pathway (i.e., local connectomes) defined by the density of the diffusing spins. Connectometry tracks the segments of a white matter fascicle that exhibits significant association with the study variable, as compared to mapping the association between a study variable and mean FA in a voxel or representing a whole tract. As the dMRI data are reconstructed into standard space and tracking is based on template, it also minimizes bias induced by manual tracking. Six multiple regression models were performed to explore local connectomes associated with (1) connected spoken language production efficacy (composite score of correct information units; *N* = 45), (2) connected singing efficacy (*N* = 43), (3) connected singing in high spoken language production efficacy group (*N* = 22), (4) connected singing in low spoken language production efficacy group (*N* = 21), (5) spoken repetition efficacy (*N* = 45) and (6) sung repetition efficacy (*N* = 45). QA-values were normalised to homogenize the data to account for two scanning sites. Age and lesion volume were controlled as covariates in all diffusion measures^69^. Due to set Bonferroni correction (*p* < 0.0083, see below) and the lowest degrees of freedom = 18 in the analysis of connected singing in low spoken language production efficacy group, conversion of *p*-value to *t*-value (two-tailed) equals to 2.96. Therefore, QA-aided deterministic fibre tracking algorithm^28^ at a T-score threshold of 3 was used to track trajectories and obtain correlational tractography. Topology-informed pruning^70^ with 4 iterations was applied to filter false fibre trajectories. Tracks with a length threshold exceeding 30 voxel distance were selected. Two thousand randomized permutations were run to obtain the null distribution of the track length and subsequently estimate the false discovery rates (FDR). The pattern of results (i.e., significant tracts or tract segments) were inspected and tracts constituting of single spurious fibres likely to be identified due to statistical noise were removed, and robust tract segments were separated. Separate robust tract segments were first identified by an automated method implemented in the DSI Studio and then checked visually by two authors (A.P. and A.J.S.) separately. When necessary, the Human Connectome Project template HCP 1065 -based tracking was consulted to make sure the correct fibre bundle for classification. Furthermore, for reporting purposes, the tracts were grouped based on the nomenclature presented in the Atlas of Human Brain Connections^71^, but referred to as dorsal stream when belonging to the perisylvian network (arcuate fasciculus) and ventral stream when belonging to the inferior network (inferior longitudinal fasciculus, inferior fronto-occipital fasciculus and uncinate).

Due to six calculated regression models, FDR threshold was set to 0.0083 (Bonferroni correction). To control for the effects of additional confounding factors (i.e., comorbid speech motor difficulties, time since stroke, choir singing experience or amusia), connectometry analyses for connected spoken language production and connected singing were reproduced with each of these measures as a third covariate (Supplementary Fig. 1).

### Reporting summary

Further information on research design is available in the Nature Portfolio Reporting Summary linked to this article.

## Supplementary information

Supplementary Information

Reporting Summary

## Data Availability

Anonymised data reported in this manuscript are available from the corresponding author upon reasonable request and subject to approval by the appropriate regulatory committees and officials. No custom codes were used in the analysis of this study. All software and packages, their versions and relevant specifications are stated in the Methods section.
