# Supplementary material for: Hodological organization of spoken language production and singing in the human brain
Source: Commun Biol. 2023 Jul 26;6:779. doi: 10.1038/s42003-023-05152-y (PMC10371982; doi:10.1038/s42003-023-05152-y)
Supplement: Supplementary file 1 — Supplementary Information [file 42003_2023_5152_MOESM1_ESM.pdf]

Supplementary information
for
Hodological organization of spoken language production and singing in the human brain

Anni Pitkäniemi, Teppo Särkämö, Sini-Tuuli Siponkoski, Sonia L.E. Brownsett, David A. Copland, Viljami Sairanen, and Aleksi J. Sihvonen

Contents

Supplementary Figure 1. Secondary analyses of connected spoken language production and singing efficacy controlling for additional confounding factors

Supplementary Figure 2. White matter connectome negatively associated with connected singing efficacy in more severe spoken language deficits group

Supplementary Figure 3. Registration result of the orientation distribution function to MNI space for each patient (N = 45)

Supplementary Figure 4. Lesion overlap map for all participants (N = 45)

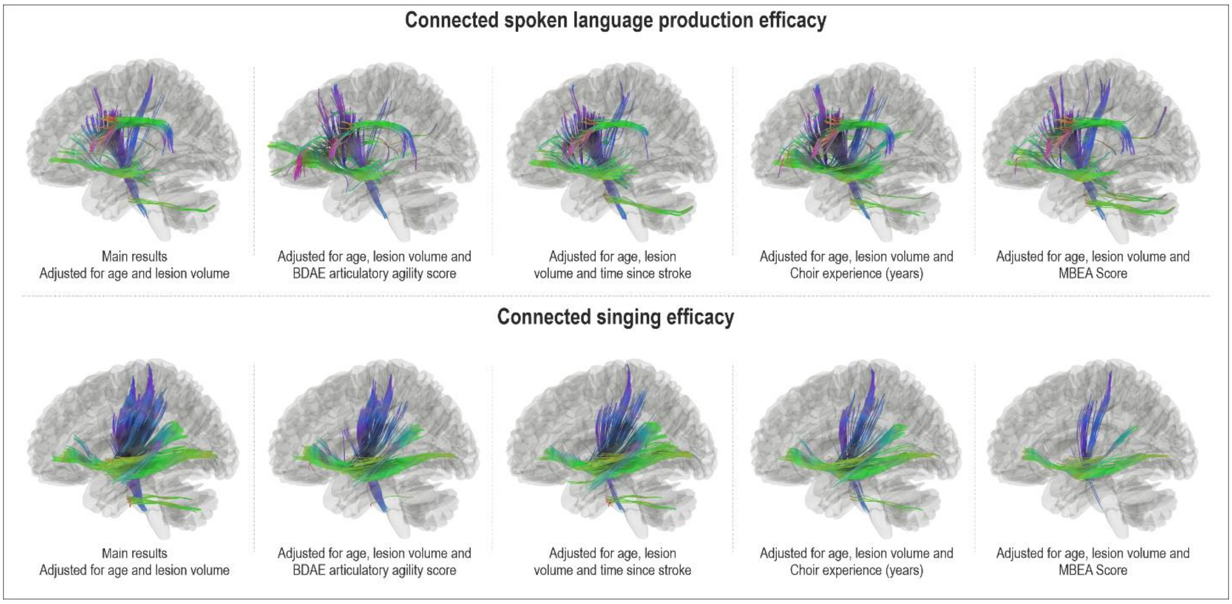

Supplementary Figure 1. Secondary analyses of connected spoken language production and singing efficacy controlling for additional confounding factors. Main results controlled for additional confounding factors (alongside age and lesion volume). For all analyses, T-score threshold = 3, voxel length threshold = 30 and false discovery rate (FDR) = 0.0083.

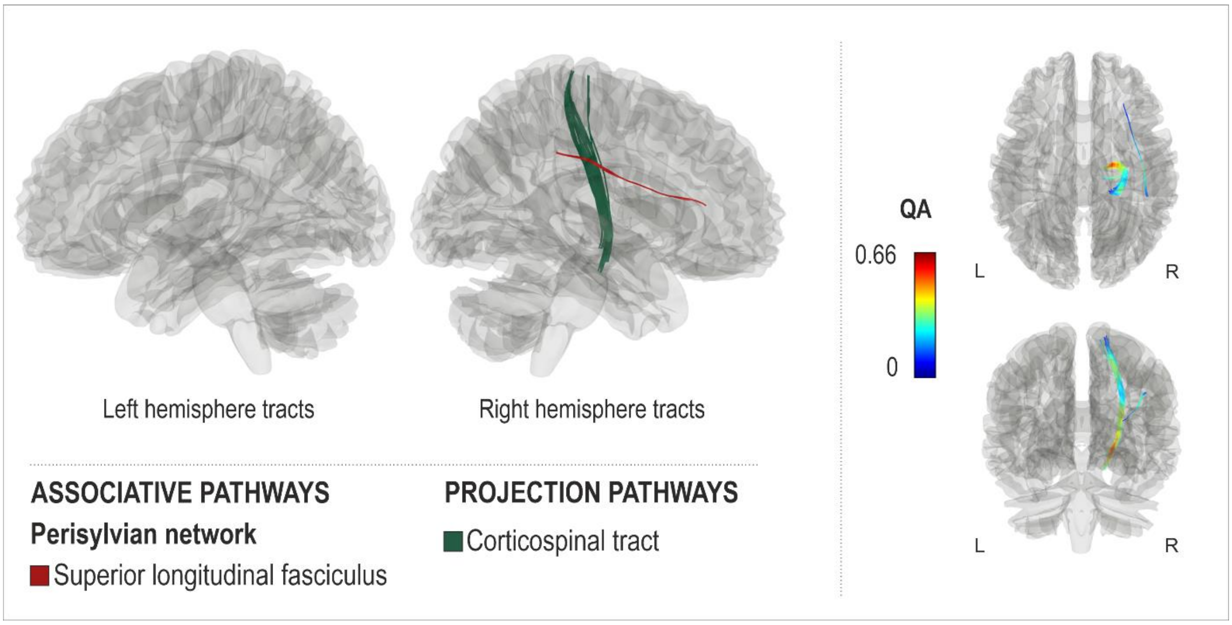

Supplementary Figure 2. White matter connectome negatively associated with connected singing efficacy in more severe spoken language deficits group. In the more severe spoken language deficits group (n = 21), poorer connected singing efficacy was associated with greater QA in the right dorsal stream (superior longitudinal fasciculus) and right projection pathways (corticospinal tract) (FDR < 0.0083). L = left, R = right.

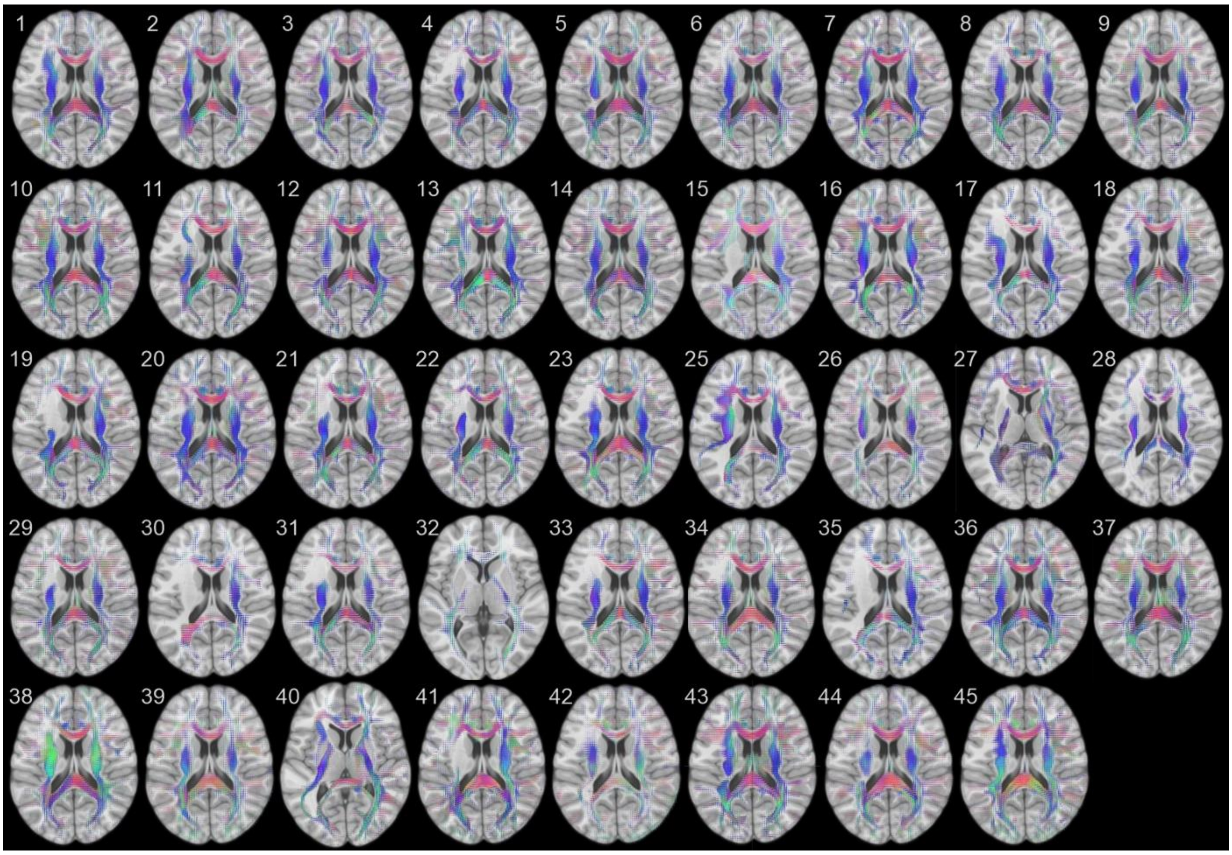

Supplementary Figure 3. Registration result of the orientation distribution function to MNI space for each patient (N = 45).

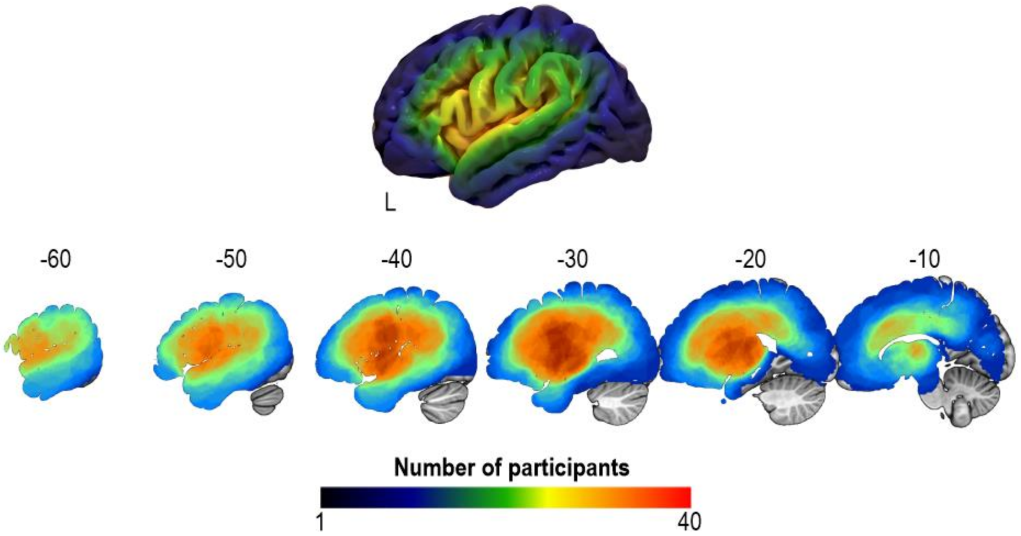

Supplementary Figure 4. Lesion overlap map for all participants (N = 45). The warmer the areas, the greater the lesion overlap. Accordingly, the color scale ranges from 1 to 40 overlapping participants. Left hemisphere is shown with MNI coordinates at the top of each slice.
