# Supplementary material for: Hodological organization of spoken language production and singing in the human brain
Source: Commun Biol. 2023 Jul 26;6:779. doi: 10.1038/s42003-023-05152-y (PMC10371982; doi:10.1038/s42003-023-05152-y)
Supplement: Supplementary file 2 — Reporting Summary [file 42003_2023_5152_MOESM2_ESM.pdf]

nature portfolio

Corresponding author(s): Anni Pitkäniemi

Last updated by author(s): Jul 7, 2023

Reporting Summary

Nature Portfolio wishes to improve the reproducibility of the work that we publish. This form provides structure for consistency and transparency in reporting. For further information on Nature Portfolio policies, see our Editorial Policies and the Editorial Policy Checklist.

Statistics

For all statistical analyses, confirm that the following items are present in the figure legend, table legend, main text, or Methods section.

n/a	Confirmed	
☐	☒	The exact sample size (*n*) for each experimental group/condition, given as a discrete number and unit of measurement
☐	☒	A statement on whether measurements were taken from distinct samples or whether the same sample was measured repeatedly
☐	☒	The statistical test(s) used AND whether they are one- or two-sided<br>*Only common tests should be described solely by name; describe more complex techniques in the Methods section.*
☐	☒	A description of all covariates tested
☐	☒	A description of any assumptions or corrections, such as tests of normality and adjustment for multiple comparisons
☐	☒	A full description of the statistical parameters including central tendency (e.g. means) or other basic estimates (e.g. regression coefficient) AND variation (e.g. standard deviation) or associated estimates of uncertainty (e.g. confidence intervals)
☐	☒	For null hypothesis testing, the test statistic (e.g. *F*, *t*, *r*) with confidence intervals, effect sizes, degrees of freedom and *P* value noted<br>*Give P values as exact values whenever suitable.*
☒	☐	For Bayesian analysis, information on the choice of priors and Markov chain Monte Carlo settings
☒	☐	For hierarchical and complex designs, identification of the appropriate level for tests and full reporting of outcomes
☒	☐	Estimates of effect sizes (e.g. Cohen's *d*, Pearson's *r*), indicating how they were calculated

Our web collection on statistics for biologists contains articles on many of the points above.

Software and code

Policy information about availability of computer code

Data collection	No custom codes were used
Data analysis	No custom codes were used

For manuscripts utilizing custom algorithms or software that are central to the research but not yet described in published literature, software must be made available to editors and reviewers. We strongly encourage code deposition in a community repository (e.g. GitHub). See the Nature Portfolio guidelines for submitting code & software for further information.

Data

Policy information about availability of data

All manuscripts must include a data availability statement. This statement should provide the following information, where applicable:

- Accession codes, unique identifiers, or web links for publicly available datasets
- A description of any restrictions on data availability
- For clinical datasets or third party data, please ensure that the statement adheres to our policy

Anonymised data reported in this manuscript are available from the corresponding author upon reasonable request and subject to approval by the appropriate regulatory committees and officials.

Human research participants

Policy information about studies involving human research participants and Sex and Gender in Research.

Reporting on sex and gender	We report sex
Population characteristics	Forty-five participants (25 females, mean age = 64.4, SD = 10.2) with post-stroke aphasia participated in this study. All participants had chronic aphasia (>6 months since onset) due to left hemisphere stroke, were over 18 years and spoke Finnish as a native language and had no hearing deficits, severe cognitive impairments (affecting their ability to co-operate and provide an informed consent), substance abuse or neurological or psychiatric comorbidities.
Recruitment	Participants were recruited from Helsinki and Turku regions in Finland during 2017-2019 through patient organizations (Helsinki-Uusimaa and Turku region Stroke Association, Finnish Brain Association) and clinical speech therapists.
Ethics oversight	The study was approved by the Ethics Committees of the Hospital Districts of Helsinki-Uusimaa and Southwest Finland.

Note that full information on the approval of the study protocol must also be provided in the manuscript.

Field-specific reporting

Please select the one below that is the best fit for your research. If you are not sure, read the appropriate sections before making your selection.

☒ Life sciences ☐ Behavioural & social sciences ☐ Ecological, evolutionary & environmental sciences

For a reference copy of the document with all sections, see nature.com/documents/nr-reporting-summary-flat.pdf

Life sciences study design

All studies must disclose on these points even when the disclosure is negative.

Sample size	A sample of 45 participants should provide power of at least 0.8 to detect relationships between language skills and white matter connectivity patterns as suggested by previously reported data on white matter connectivity in post-stroke aphasia.
Data exclusions	If the behavioural outcome (performance measure) was missing, participant was excluded from the analysis in question. On the connected singing task, the digital recordings of two participants were lost due to technical malfunction. There were no other missing data.
Replication	Main analyses were replicated with secondary analyses controlling for additional confounding factors. Results are reported in Supplementary Information (Supplementary Figure 1)
Randomization	No group allocation
Blinding	Not relevant

Reporting for specific materials, systems and methods

We require information from authors about some types of materials, experimental systems and methods used in many studies. Here, indicate whether each material, system or method listed is relevant to your study. If you are not sure if a list item applies to your research, read the appropriate section before selecting a response.

Materials & experimental systems

n/a	Involved in the study	
☒	☐	Antibodies
☒	☐	Eukaryotic cell lines
☒	☐	Palaeontology and archaeology
☒	☐	Animals and other organisms
☒	☐	Clinical data
☒	☐	Dual use research of concern

Methods

n/a	Involved in the study	
☒	☐	ChIP-seq
☒	☐	Flow cytometry
☐	☒	MRI-based neuroimaging

Magnetic resonance imaging

Experimental design

Design type	n/a
Design specifications	n/a
Behavioral performance measures	Language and singing outcomes

Acquisition

Imaging type(s)	Structural, diffusion
Field strength	3T
Sequence & imaging parameters	Repetition time (TR) = 5000 ms, echo time (TE) = 104 ms, field of view (FOV) = 240 x 240 mm, voxel size 2.0 x 2.0 x 2.0 mm3.
Area of acquisition	Whole brain

Diffusion MRI ☒ Used ☐ Not used

Parameters: Multi-shell diffusion-weighted MRI (DW-MRI) with 13 non-diffusion weighted volumes and 130 diffusion weighted volumes (30 volumes with b = 1000 s/mm2 and 100 volumes with b = 2500 s/mm2)

Preprocessing

Preprocessing software	MRTrix3, DSI Studio
Normalization	Normalized using default DSI Studio's QSDR reconstruction protocol that combines both linear and nonlinear approaches using b0-images and diffusion images.
Normalization template	1-mm MNI space, HCP1021-template
Noise and artifact removal	The DW-MRI data were denoised using Marchenko-Pastur PCA method. Gibbs ringing artefact correction was performed with a method based on local subvoxel-shifts.
Volume censoring	n/a

Statistical modeling & inference

Model type and settings	Multiple regression
Effect(s) tested	Local connectomes associated with 1) connected spoken language production efficacy (composite score of correct information units), 2) connected singing efficacy, 3) connected singing in high spoken language production efficacy group, 4) connected singing in low spoken language production efficacy group, 5) spoken repetition efficacy and 6) sung repetition efficacy.

Specify type of analysis: ☒ Whole brain ☐ ROI-based ☐ Both

Statistic type for inference (See Eklund et al. 2016)	Thresholds: T-score≥3, voxel length≥30. Topology-informed pruning with 4 iterations was applied to filter false fiber trajectories.
Correction	Bonferroni

Models & analysis

n/a	Involved in the study
☒	☐ Functional and/or effective connectivity
☒	☐ Graph analysis
☒	☐ Multivariate modeling or predictive analysis
